# Cephalometric comparison of Eastman standards among caucasians and saudi population in the Najran region

**DOI:** 10.6026/973206300181173

**Published:** 2022-12-31

**Authors:** Mohammed Alassiry Ahmed

**Affiliations:** 1 Department of Preventive Dental Sciences, Faculty of Dentistry, Najran University, Najran, Saudi Arabia

**Keywords:** Cephalometrics, Saudi Arabia, Caucasian, Najran, Eastman standards

## Abstract

Caucasian norms derived from the European-American population are often applied in the diagnosis and treatment planning of Saudi orthodontic patients. Minor differences in cephalometric values can be considered routine and normal, but significant differences indicate a structural deviation. The objective of this study was to establish the cephalometric norms of normal and pleasant soft tissue profiles of adult Saudi living in the Najran region of Saudi Arabia and compare them with the Eastman standards derived from the Caucasian population. A total of 60 lateral cephalometric radiographs (30 males and 30 females) of Najran adults aged between 18-30 years with pleasing, acceptable, harmonious and well-balanced soft tissue profile and Class I molar relationship with no history of previous orthodontic treatment, no crowding or spacing, normal overjet and overbite were selected for the study. Descriptive analysis and Student t-test were performed to evaluate the male and female gender differences and compare the Najran mean with Eastman standards. On evaluating the gender differences, Najran females tend to have more ANB angle and L1 to A-Pog linear distance than Najran males(p<0.05). On comparing with the Eastman Caucasian standards, the Najran population tends to have more SNA, ANB, UI to MxP and L1 to A-Pog (p<0.05). The U1 to L1 angle tends to be less in Najran population than the Caucasians (p<0.05). The Najran population has more protrusive skeletal bases, bimaxillary protrusion, more proclined upper and lower incisors and reduced interincisal angle than Caucasians. The Najran females have more protrusive skeletal bases and more proclined lower incisors compared to Najran males. The cephalometric findings of this study can be useful in the diagnosis and treatment planning of orthodontic patients belonging to the Najran region of Saudi Arabia.

## Background:

In orthodontics, there's a plenitude of cephalometric analysis to identify problems of craniofacial aspects in a patient's anatomy. [1]Since most cephalometric standards are derived from the Caucasian population, they've been consistently used in non-Caucasian populations for various research and diagnostic purposes. [2] Various studies have established that it is inappropriate to applystandard values derived from a single population to other diverse populations, racial and ethnic groups or subgroups. [3] Eastman standards were derived from the work of Ballard (1948), Mills(1982) and MacAllister and Rock (1992). [4, 5] Although Saudi Arabians are considered a subgroup of Caucasians, at the current time, Saudi Arabia consists of a population of mixed ethnic origin.[6] This can be attributed to an amalgamation of different communities owing to the presence of Islamic pilgrimage, oilrelated immigration and Afro-Asian circle. [7] Orthodontic literature consists of many studies comparing cephalometric standards of the Saudi and Arab populations with Caucasian standards [6-12]. There is an agreement that the Saudi population demonstrates greater proclination and protrusion of the dentoalveolar complex compared to the Caucasian population. Lamentably, previous studies have been performed in the central, western and Riyadh regions of Saudi Arabia. Till date, no study has been performed on the population of Najran in Saudi Arabia. The objective of this study is to analyze and establish cephalometric norms ('Najran Standards') of normal and pleasant soft tissue profiles of adult Saudi males and females in the Najran region and compare them with Eastman Caucasian standards.

## Material and Methods:

The sample consisted of 60 lateral cephalometric radiographs (30males and 30 females) of Saudi adults aged between 18-30 years living in the Najran region of Saudi Arabia. The study was approved by Najran University's ethical committee (441-40-57548). The patient visited the Sama dental centre for an orthodontic consultation and a lateral cephalogram was necessary for diagnosis purposes were used for the current study by using the following inclusion criteria:

[1] Radiographs in the age group of 18-30 years Saudis residing in Najran only.

[2] Pleasing, harmonious soft tissue profile and well-balanced face as perceived on lateral cephalograms.

[3] Class I molar and canine relationship with normal overjet (< 4mm) and overbite (5-20%).

[4] Minimal or no crowding or spacing.

[5] Presence of permanent dentition with no missing permanent teeth except third molars.

[6] No history of previous orthodontic treatment.

[7] No history of trauma, fractures, craniofacial malformations and syndromes.

l[8] Good quality lateral cephalograms with no distortion and proper orientation of the head.

The cephalograms were taken on Veraview FX800 (Morita, California, USA). The cephalograms which were deemed appropriate were included in the final sample. The final sample of 60 cephalograms was digitally analyzed using Dolphin Imaging Software Version 10.5 (Dolphin Imaging and Management Solutions, Chatsworth, California, USA). Eastman cephalometric analysis [5] consisting of 8 angular measurements and 1 linear measurement was performed (Figure 1-see PDF). The digital analysis was performed by a single investigator by clearly identifying anatomical landmarks of the cephalograms directly on the monitor and placing a dot over it. Once all required anatomical landmarks were identified, the software automatically generated angular and linear measurements for a single cephalogram. The data were recorded and tabulated in Excel (Version:2003, Microsoft, Redmond, USA). An intra-examiner test was performed to assess the reliability of the measurements. Around 15 cephalograms were randomly selected from the final sample and were re-recorded again after two weeks intervals by the same investigator. Dahlberg's formula and coefficient of reliability (ICC) were used to calculate the correlation between two sets of readings. The Caucasian data were used from previous studies to compare it with the data of the Saudi population from the Najran region. The data was statistically analysed using SPSS, Version 20 (IBM Corp., Armonk, New York, USA). Descriptive analysis was used to obtain the mean, standard deviation, and different values of the male and female samples of the Najran population. Independent Student's t-test was used to evaluate gender differences and compare the mean values of the Najran population with values of Eastman standards. A p-value of <0.05 was considered to be statistically significant.

## Results:

A total of 60 lateral cephalograms of 30 males and 30 females were evaluated. The overall mean age of the sample was 24.74±5.08 years. The mean age of the males was 25.30±5.60 years and for females were 24.18±5.09 years. The Dahlberg's error ranged from 0.0 to 0.65 degrees for angular measurements and 0.0 to 0.36 mm for linear measurements. Inter-class correlation coefficient (ICC) found a high correlation (>10.9) between the two sets of readings. No statistical difference was found in the two sets of readings (p>0.05). The descriptive statistics and gender differences of the Najran population are shown in Table 1(see PDF). Only two parameters, ANB and L1 to A-Pog were found statistically significant (p<0.05) between the male and female populations. The ANB in females (5.23°±1.79°) was larger in comparison to males (3.99°±2.33°) by 1.24° and L1 to A-Pog linear distance was more in females (4.22 mm±1.97 mm) as compared to males (2.97 mm±2.02 mm) by 1.25 mm. Remaining other parameters, the difference was found to be statistically insignificant between the male and female populations. In this study, Najran standards were compared with Eastman standards shown in Table 2. Only SNA, ANB, UI to MxP, U1 to L1 and L1 to APog were statistically significant (p<0.05).SNA angle was more in the Najran population (82.85°±3.15°) compared to Caucasians (81°±3°) by 1.85°. ANB angle was more in the Najran population (4.61°±2.16°) compared to Caucasians (3°±2°) by 1.61°. UI to MxP angle was more in the Najran population (114.8°±5.29°) compared to Caucasians (109°±6°) by 5.8°. U1 to L1 angle was more in Caucasians (123.9°±8.58°) compared to the Najran population (131°±6°) by 7.1°. L1 to APog linear distance was more in the Najran population (3.6 mm±2.08 mm) compared to Caucasians (1 mm±2 mm) by 2.6 mm.

## Discussion:

Cephalometric analysis is important for optimum orthodontic diagnosis and treatment planning. Many studies in past have proved that cephalometric norms derived from a particular ethnic group might not be necessarily relevant to others. [3,13] This holds equally true for the Arabic population which is considered a truly ethnically diverse composite of several races. A recent meta-analysis concluded that the Arab population doesn't constitute a homogeneous ethnic entity. [14] Considering the above disparity, this study has been done to exclusively establish cephalometric norms of normal and pleasant soft tissue profiles of the adult population of the Najran region in Saudi Arabia. In this study, the SNA angle has been found to be more and statistically significant in the Najran population (82.85°±3.15°) compared to Caucasians (81°±3°). Thus, it can be inferred that the Najran population presents with more prognathic maxilla compared to Caucasians. Similarly, the SNB angle was marginally larger but statistically insignificant in the Najran population (78.24°±2.83°) compared with Caucasians (78°±3°). ANB angle was more in the Najran population (4.61°±2.16°) when compared to Caucasians (3°±2°) and was statistically significant. Thus, it can be concluded that the Najran population presents with more protrusive skeletal bases and bimaxillary protrusion. This is in accordance with the findings of Shalhoub, [8] Sarhan and Nashashibi, [9] Nashashibi, [10] Al-Jasser [6] and Hassan [7] who performed their studies on the Saudi population. Shalhoub [8] found that Saudi female demonstrates a protrusive maxilla whereas Saudi males present with a more protrusive midface. Sarhan and Nashashibi [9] found that the SNA, SNB and ANB were larger in the Saudi population compared to the British population but were found to be non-significant. Similarly, Nashashibi [10] revealed a more protrusive maxillary apical base and bimaxillary protrusion. Al-Jasser [6] concluded that adult Saudis demonstrated more bimaxillary protrusion compared to Caucasians. Hassan [7] found that Saudis tend to have increased ANB and bimaxillary protrusion compared to Caucasians.

On evaluating the dental parameters in this study, the interincisal (U1 to L1) angle was more acute in the Najran population (123.9°±8.58°) than in Caucasians (131°±6°) indicating proclined upper and lower incisors. The UI to MxP angle was more in the Najran population (114.8°±5.29°) than Caucasians (109°±6°) specifying more proclined upper incisors. Also, the L1 to A-Pog linear distance was more in the Najran population (3.6 mm±2.08 mm) than Caucasians (1 mm±2 mm) suggesting more proclination of lower incisors. This clearly indicates that incisors are significantly more proclined and protruded in the Najran population as compared to Caucasians. This is in agreement with the findings of Sarhan and Nashashibi, [9] Al-Jasser [6] and Hassan [7] who have consistently reported increased proclination of upper and lower incisors and reduced interincisal angle in the Saudi population compared to the Caucasians. Further, the maxillary mandibular plane angle (MMPA) was 26.76°±5.16° in the Najran population compared to 27°±4° in Caucasians (Table 2). The findings of this study are not in agreement with the studies conducted by Al-Jasser and Hassan. [6-7] They both found steeper MMPA in Saudis compared to Caucasians. Hence the population of Najran demonstrates MMPA which is less than other studied populations of Saudi Arabia but similar to Caucasian norms. On evaluating the gender differences, the ANB angle in females (5.23°±1.79°) was larger than males (3.99°±2.33°) and the L1 to A-Pog linear distance was more in females (4.22 mm±1.97 mm) compared to males (2.97 mm±2.02 mm) (Table 1-see PDF). Thus, the Najran females present with more protrusive skeletal bases and more proclined lower incisors in relation to the A-Pog line. Hassan [7] found that Saudi males tend to have more prognathic mandible (increased SNB) than females. The other parameters evaluated in this study have been found to be statistically insignificant between males and females. In comparison with other world populations with Caucasians, Bishara [15] reported dental protrusion in the Egyptian population compared to Iowa samples. Handan and Rock [16] found that the Jordanian population presented with proclined incisors with reduced interincisal angle and significantly forward-placed lower incisors in relation to the A-Pog line. Behbehani [17] found that Kuwaitis had more facial convexity and dental protrusion along with retruded mandibles. A recent study concluded that Emiratis present with greater incisor proclination and bimaxillary protrusion than Caucasians. [18] In this study, only those cephalograms of a patient who desired to seek orthodontic treatment were evaluated. This can attribute to the finding of greater proclination and protrusion in this study. Hence, a larger sample with different age groups, more variables and stricter criteria can be considered for further investigations.

## Conclusion:

In comparison to Eastman Caucasian standards, the Najran populations have protrusive skeletal bases, bimaxillary protrusion, proclined upper and lower incisors and reduced interincisal angle. The Najran population has normal MMPA compared to Caucasians but less than other populations in Saudi Arabia. The Najran females have more protrusive skeletal bases and more proclined lower incisors compared to Najran males. The authors recommend the use of race-specific cephalometric norms for the evaluation of particular individuals from a distinct population. The Najran standards presented in this study can be of value in the cephalometric evaluation of individuals belonging to this region.

## References

[1] Brodie AG (1949). J Oral Surg..

[2] Flynn TR (1989). J Oral Maxillofac Surg..

[3] Cotton WN (1951). Angle Orthod..

[4] Ballard CF (1956). Transactions of the European Orthodontic Society..

[5] MacAllister MJ (1992). Br J Orthod.

[6] Al-Jasser NM (2005). J Contemp Dent Pract..

[7] Hassan AH (2006). Angle Orthod..

[8] Shalhoub SY (1987). Br J Orthod..

[9] Sarhan OA (1988). J Rehab..

[10] Nashashibi IA (1990). The Saudi Dent J..

[11] Al-Jasser NM (2000). Saudi Med J..

[12] Al-Jasser NM (2003). Saudi Med J..

[13] Kowaliski CJ (1974). Angle Orthod..

[14] Alshammery DA (2016). Saudi J Oral Sci..

[15] Bishara SE (1990). Am J Orthod Dentofacial Orthop..

[16] Hamdan AM (2001). J Orthod..

[17] Behbehani F (2006). Angle Orthod..

[18] Al Zain T (2012). J Orthodont Sci..

